# Derivatives of phenyl tribromomethyl sulfone as novel compounds with potential pesticidal activity

**DOI:** 10.3762/bjoc.8.27

**Published:** 2012-02-15

**Authors:** Krzysztof M Borys, Maciej D Korzyński, Zbigniew Ochal

**Affiliations:** 1 Faculty of Chemistry, Warsaw University of Technology, Noakowskiego 3, Warsaw 00-664, Poland

**Keywords:** 2-nitroaniline derivatives, phenylhydrazones, pesticides, S_N_Ar reaction, tribromomethyl sulfone derivatives

## Abstract

A halogenmethylsulfonyl moiety is incorporated in numerous active herbicides and fungicides. The synthesis of tribromomethyl phenyl sulfone derivatives as novel potential pesticides is reported. The title sulfone was obtained by following three different synthetic routes, starting from 4-chlorothiophenol or 4-halogenphenyl methyl sulfone. Products of its subsequent nitration were subjected to the S_N_Ar reactions with ammonia, amines, hydrazines and phenolates to give 2-nitroaniline, 2-nitrophenylhydrazine and diphenyl ether derivatives. Reduction of the nitro group of 4-tribromomethylsulfonyl-2-nitroaniline yielded the corresponding *o*-phenylenediamine substrate for preparation of structurally varied benzimidazoles.

## Introduction

The rapid growth of the world population results in a continous increase in the demand for food. At the same time, about 35% of the global crops around the world are being destroyed due to a diverse range of diseases as well as a wide variety of pests and weeds [1]. Both of these factors are nowadays the reason for the growing interest in the development of new, selective and efficient pesticides. Maintaining our investigations into the preparation of novel pesticidal agents, we turned our attention to compounds possessing a halogenmethylsulfonyl group. Aromatic compounds featuring this moiety has been reported to exhibit the desired biological activity [2–7]. The results of our previous research revealed that the presence of halogenmethylsulfonyl groups in some nitroaniline and benzimidazole derivatives was beneficial to their herbicidal and fungicidal activity [6,8–9]. Moreover, some 2-nitroaniline and 2,6-dinitroaniline derivatives belong to the group of commonly applied herbicides [10].

**Scheme 1:**
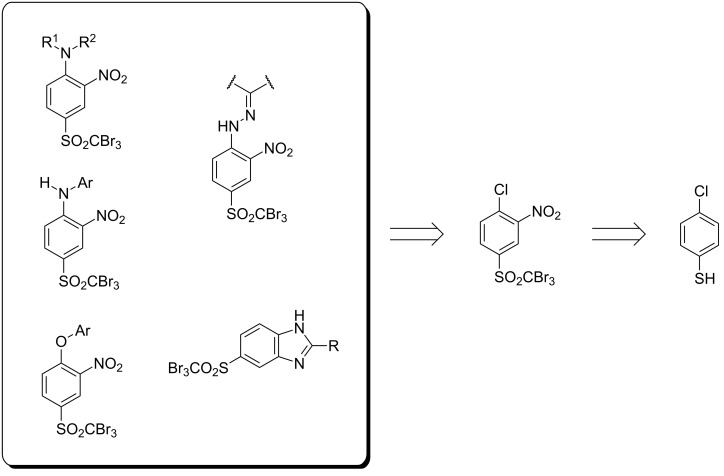
Retrosynthetic analysis of the designed target molecules.

Herein we report the synthesis of novel aromatic compounds containing a tribromomethylsulfonyl group, including derivatives of nitroaniline, nitrophenylhydrazine, diphenyl ether and benzimidazole (Scheme 1). All the target molecules are derived from a common precursor, namely 4-halogenphenyl tribromomethyl sulfone, efficiently synthesized from inexpensive 4-halogenthiophenol. The development of an effective method for the preparation of the title compounds allowed an evaluation of their biological activity against certain fungi to be carried out.

## Results and Discussion

**Scheme 2:**
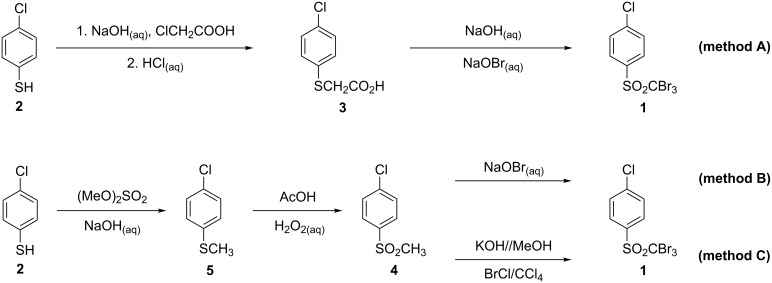
Synthetic routes for 4-chlorophenyl tribromomethyl sulfone (**1**).

Our initial synthetic efforts were aimed at the preparation of the starting compound 4-halogenphenyl tribromomethyl sulfone (where halogen stands for chlorine or bromine). 4-Chlorophenyl tribromomethyl sulfone (**1**) was obtained by following three different synthetic methods (Scheme 2).

The first approach utilized the method for trihalogenmethyl sulfone synthesis developed by Farrar [11] (Scheme 2, method A). Reaction of 4-chlorothiophenol (**2**) with sodium hydroxide and sodium chloroacetate (obtained in situ by neutralization of chloroacetic acid with NaOH solution) afforded 2-(4-chlorophenylthio)acetic acid (**3**). Oxidative bromination of **3** with sodium hypobromite gave sulfone **1** in moderate (57%) overall yield, the second step being particularly time-consuming (80 h). These significant drawbacks led us search for other preparation methods. We considered that the readily available methylsulfone **4** may serve as an intermediate in the synthesis of **1** (Scheme 2, methods B and C). It was obtained by *S*-methylation of 4-chlorothiophenol (**2**) with dimethyl sulfate, followed by treatment of the resulting product **5** with hydrogen peroxide and glacial acetic acid. Next, bromination of the methyl group of **4** was achieved by using either sodium hypobromite (Scheme 2, method B) or bromine chloride (Scheme 2, method C), both of which have been extensively researched by us as halogenating agents. The overall yields of tribromomethyl sulfone **1** obtained by this pathway amounted to 86% and 85% (starting from **2**) for the NaOBr and BrCl method of bromination, respectively.

**Scheme 3:**
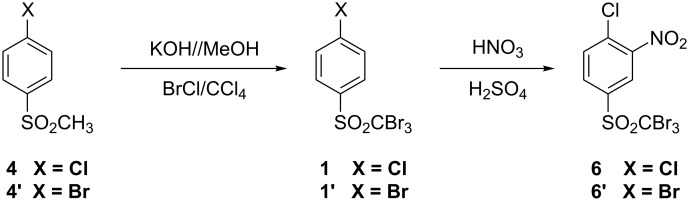
Halogenation/nitration sequence for 4-halogenphenyl methyl sulfones **4** and **4'**.

The next step in the functionalization of sulfone **1** was its nitration, carried out by standard means of a mixture of concentrated nitric and sulfuric acids (Scheme 3) [12].

As pesticides are chemicals produced most commonly in considerable quantities, optimization of the synthetic process is highly desirable. Bearing in mind that the obtained nitro compound is the key intermediate for all further transformations, we decided to check whether the use of bromine-substituted aromatic methylsulfone **4'** would prove to offer higher yields in the halogenation/nitration sequence. Bromination of **4'** with BrCl afforded tribromomethyl sulfone **1'** in 90% yield (compared to 94% for chlorine analogue **1**), while the subsequent nitration of **6'** resulted in 94% yield (compared to 96% for analogue **6**). With these results at hand, we ultimately picked chlorine-containing nitrosulfone **6** as the substrate for the subsequent synthetic steps.

**Scheme 4:**
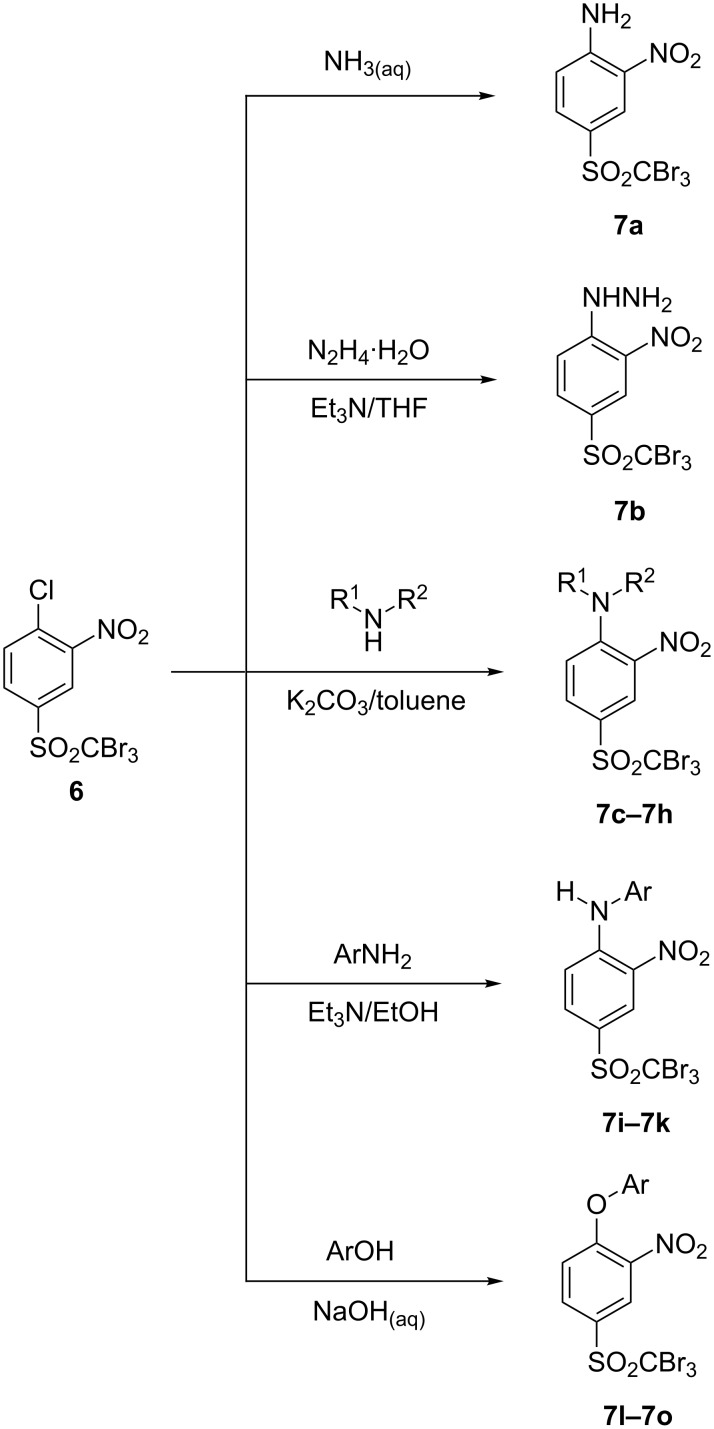
S_N_Ar transformations of sulfone **6**.

A range of S_N_Ar reactions of **6** with various *N*- and *O*-nucleophiles was carried out (Scheme 4).

First, aniline derivative **7a** (by treatment of **6** with aqueous ammonia) and phenylhydrazine derivative **7b** (by treatment of **6** with hydrazine hydrate and triethylamine as a coproduced HCl acceptor) were prepared. Sulfone **6** was also reacted with aliphatic amines (products **7c**–**7h**), aromatic primary amines (products **7i**–**7k**) and phenols (products **7l**–**7o**), with all the resulting compounds being obtained in >85% yields (Table 1; see Supporting Information File 1 for further details on products **7a**–**7o**).

**Table 1:** Derivatives of 2-nitroaniline, 2-nitrophenylhydrazine and diphenyl ether.

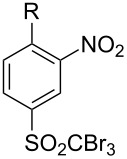		

product	substituent (R)	yield^a^ (%)

**7a**	NH_2_	93
**7b**	NHNH_2_	94
**7c**	NHCH_3_	95
**7d**	NHC_6_H_11_	87
**7e**	NHCH_2_CH_2_CH_3_	92
**7f**	NHCH_2_CH(CH_3_)_2_	94
**7g**	N(C_2_H_5_)_2_	94
**7h**	N(CH_2_CH(CH_3_)_2_)_2_	92
**7i**	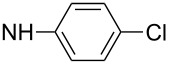	96
**7j**	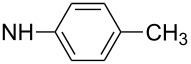	88
**7k**	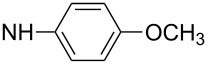	86
**7l**	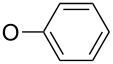	91
**7m**	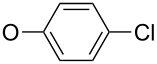	92
**7n**	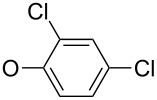	92
**7o**	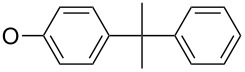	90

^a^Isolated yield.

**Scheme 5:**
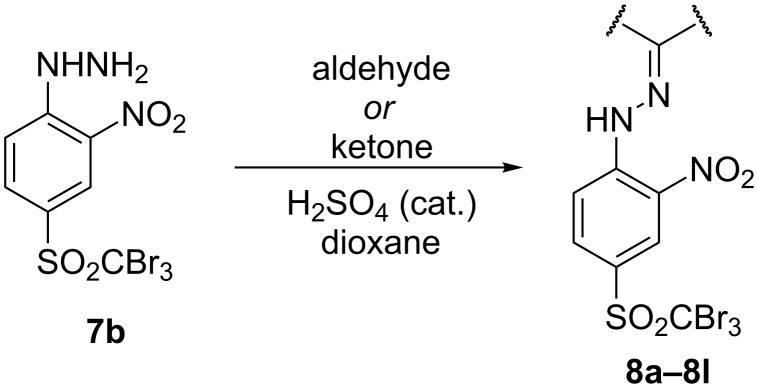
Preparation of phenylhydrazones **8a**–**8l**.

At this point, two groups of the designed target molecules remained to be synthesized: Phenylhydrazones and benzimidazoles. The acid-catalyzed reaction of phenylhydrazine derivative **7b** with aldehydes or ketones (Scheme 5) afforded a series of phenylhydrazones possessing various substituents (Table 2; see Supporting Information File 1 for further details on products **8a**–**8l**).

**Table 2:** 2-Nitro-4-tribromomethylsulfonylphenylhydrazones.

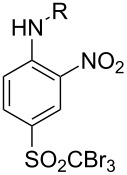		

compound	substituent (R)	yield^a^ (%)

**8a**	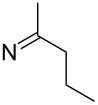	96
**8b**	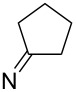	87
**8c**	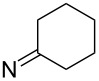	89
**8d**	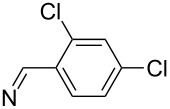	93
**8e**	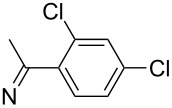	90
**8f**	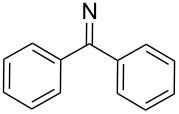	92
**8g**	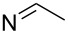	85
**8h**	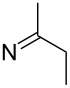	89
**8i**	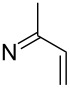	91
**8j**	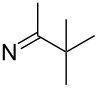	88
**8k**	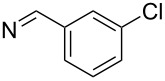	92
**8l**	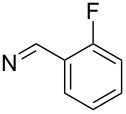	94

^a^Isolated yield.

**Scheme 6:**
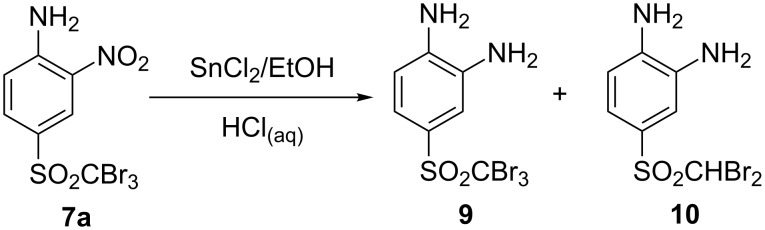
Products of the nitro group reduction of sulfone **7a**.

The two-step synthesis of benzimidazoles featured aniline derivative **7a** as the starting material (Scheme 6).

The first transformation, i.e., the reduction of the nitro group, was initially intended to be completed with stannous chloride in concentrated hydrochloric acid as a reducing agent. However, this method proceeded solely to debromination, and 4-dibromomethylsulfonyl-1,2-diamine (**10**) was isolated exclusively. Bromine cleavage in phenyl tribromomethyl sulfones was previously observed also by Fields and Shechter [13], who investigated the addition of phenyl tribromomethyl sulfone to olefins. Fortunately we found out that conducting the reduction with SnCl_2_/HCl in ethanol provided the desired diamine **9**, although along with up to 40% yield of the debrominated diamine **10**. The reaction work-up involved treatment of the resulting chlorostannic acid–diamine complexes with sodium hydroxide, the particular products being later successfully isolated by recrystallization.

**Scheme 7:**
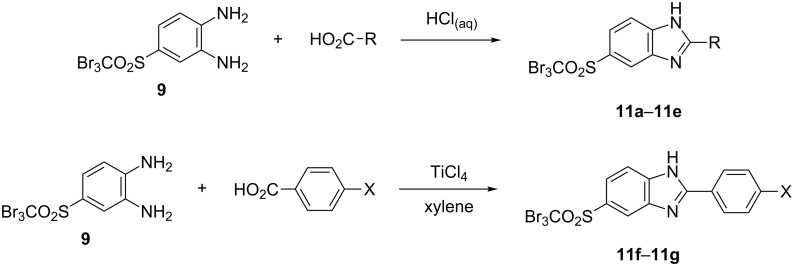
Synthesis of benzimidazole derivatives **11a**–**11g**.

With diamine **9** at hand, we proceeded to the synthesis of benzimidazole scaffolds (Scheme 7, Table 3; see Supporting Information File 1 for further details on products **11a**–**11j**).

**Table 3:** 5-Tribromomethylsulfonylbenzimidazole derivatives.

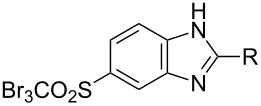		

product	substituent (R)	yield^a^ (%)

**11a**	CH_3_	86
**11b**	CF_3_	93
**11c**	CH_2_C_6_H_5_	57
**11d**	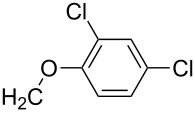	81
**11e**	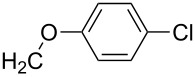	77
**11f**	C_6_H_5_	79
**11g**	C_6_H_4_-4-Cl	65
**11h**	SH	80
**11i**	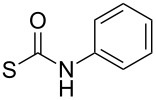	92
**11j**	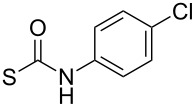	76

^a^Isolated yield.

The cyclization was carried out according to the well-established Philips method [14–15], based on treatment of the appropriate substrates with hydrochloric acid. Reactions of diamine, as well as its *alpha*-functionalized derivatives, with hydrochloric acid successfully afforded 2-substituted benzimidazoles **11a**–**11e** in moderate to high yields.

It is worth noting that we encountered significant difficulties while applying the Philips method for the cyclization of diamine **9** with benzoic acid and 4-chlorobenzoic acid. The expected benzimidazole was not formed, and only the substrates were isolated from the reaction mixture. Therefore we made an attempt at synthesizing 2-arylbenzimidazole **11f** and **11g** according to our patented method [16]. The cyclization of diamine **9** with benzoic acids was run in anhydrous xylene with titanium tetrachloride as a catalyst (Scheme 7).

**Scheme 8:**
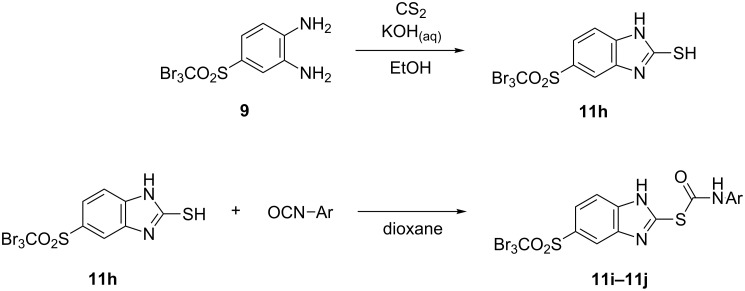
Preparation and further transformation of 2-mercaptobenzimidazole **11h**.

2-Mercaptobenzimidazole **11h** was obtained by the reaction of diamine **9** with carbon disulfide (Scheme 8) [17]. Moreover, **11h** was subjected to the reaction with aromatic isocyanates to give benzimidazolthiocarbamates **11i** and **11j**.

The synthesized compounds were tested for their fungicidal activity. The evaluation was carried out by determining the percentage inhibition of mycelium growth on agar medium under the influence of the tested compound compared with the control pan. It was found that compounds **7a**, **7m**, **7n** and **7i** exhibit high or moderate activity against some fungal pathogens (Table 4).

**Table 4:** Fungicidal activity of the active compounds^a^.

	*A. alternata*	*B. cinerea*	*F. culmorum*	*P. cactorum*	*R. solani*	*B. graminis*
	100 μg/mL	200 μg/mL 20 μg/mL				1000 μg/mL

**7a**	70	80 50	80 60	80 60	100 80	50
**7m**	80	80 40	60 0	100 20	100 30	55
**7n**	60	40 0	80 60	100 80	100 60	50
**7i**	50	80 50	80 20	70 20	90 40	35

^a^The results of biological evaluation are expressed as the values of percentage inhibition of colony growth of the fungi, derived from the following formula: *I* = 100 (*C* − *T*)/*C*, where *I* = percentage inhibition of colony growth of the fungi, *C* = zone of growth of the fungus colony in mm in the control, *T* = zone of growth of the fungus colony in mm in the examined sample. The first row of the table contains the names of the utilized fungal pathogens; the second row shows the examined concentration(s) of the compound in the sample.

## Conclusions

In conclusion, we designed and synthesized a series of tribromomethylsulfonyl bearing derivatives of 2-nitroaniline, 2-nitrophenylhydrazine, diphenyl ethers and benzimidazole. Unexpected difficulties experienced during the synthesis were successfully circumvented utilizing, among other things, our patented methodologies. The outcome of biological screening showed that some of the obtained compounds exhibit fungicidal activity.

## Supporting Information

File 1 Experimental procedures and characterization data.
