# Supplementary material for: Derivatives of phenyl tribromomethyl sulfone as novel compounds with potential pesticidal activity
Source: Beilstein J Org Chem. 2012 Feb 15;8:259–65. doi: 10.3762/bjoc.8.27 (PMC3302088; doi:10.3762/bjoc.8.27)
Supplement: File 1 — Experimental procedures and characterization data. [file Beilstein_J_Org_Chem-08-259-s001.pdf]

Supporting Information

for

Derivatives of tribromomethyl phenyl sulfone as novel compounds with potential pesticidal activity

Krzysztof M. Borys, Maciej D. Korzyński and Zbigniew Ochal*

Address: Faculty of Chemistry, Warsaw University of Technology, Noakowskiego 3, Warsaw 00-664, Poland

Email: Zbigniew Ochal - ochal@ch.pw.edu.pl

* Corresponding author

Experimental procedures and characterization data

Contents:

1. General methods (S2)
2. Experimental procedures (S2–S10)
3. Table 1. Derivatives of 2-nitroaniline, 2-nitrophenylhydrazine and diphenyl ether (S11)
4. Table 2. 2-Nitro-4-tribromomethylsulfonylphenylhydrazones (S12)
5. Table 3. 5-Tribromomethylsulfonylbenzimidazole derivatives (S13)
6. Exemplary ^{1}H and ^{13}C NMR spectra (S14–S17)

1. General methods

All reactions were set up in air, and undistilled solvents were used, unless stated otherwise. The reagents were purchased from commercial sources and were used without further purification except for Et_3N, which was distilled from potassium hydroxide prior to use. The reported yields refer to pure isolated products, unless stated otherwise. Reactions were monitored by gas chromatography (GC) and/or thin-layer chromatography (TLC) carried out on silica-gel plates by using UV light or *p*-anisaldehyde solution and heat as visualizing agents. ^{1}H NMR spectra were recorded on a Varian Mercury 400 MHz spectrometer in $CDCl_3$, using TMS (tetramethylsilane) as an internal standard; all signals are reported in ppm as (s = singlet, dd = doublet of doublets, m = multiplet, integration). IR spectra were recorded in paraffin oil on a Specord M80 Zeiss Jena spectrophotometer and reported with interpretation of significant bands. Elemental analyses were obtained by means of a Perkin Elmer 2400 apparatus. All melting points (mp) are given uncorrected.

2. Experimental procedures

2-(4-Chlorophenylthio)acetic acid (3)
(Method A)

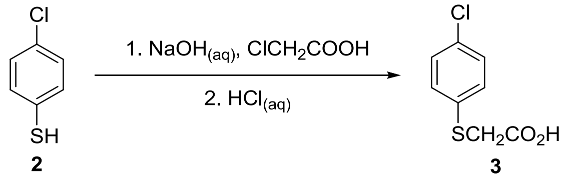

To a stirred solution of 50% w/w aq NaOH (10.9 mL) and 4-chlorothiophenol (46.0 g, 0.32 mol) sodium chloroacetate (prepared by neutralization of chloroacetic acid (30.2 g, 0.32 mol) with 50% w/w aq NaOH) was added. The mixture was heated under reflux for 2 h. After cooling of the mixture, the precipitate was filtered off, washed with 10% w/w aq NaOH saturated with sodium chloride, then dissolved in hot water and acidified with hydrochloric acid. The resulting precipitate was filtered off, washed with cold water and dried. Product **3** was obtained in 75% yield (48.0 g, 0.24 mol); mp 99–100 °C; ^{1}H NMR (400 MHz, $CDCl_3$) δ 8.02–7.96 (m, 2H), 7.68–7.62 (m, 2H), 3.24 (s, 2H).

1-Chlorophenyl-4-tribromomethylsulfone (1)
(Method A)

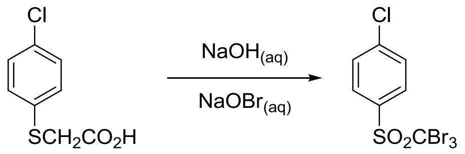

3 1

To 2-(4-chlorophenylthio)acetic acid (**3**) (10.2 g, 0.05 mol) dissolved in 0.5 M aq NaOH (150 mL), freshly prepared 14% w/w aq sodium hypobromite (250 mL) was added. The solution was stirred at room temperature for 80 hours. The resulting precipitate was filtered off, washed with water, dried and recrystallized from ethanol. Sulfone **1** was obtained in 76% yield (16.2 g, 0.038 mol); mp 144–145 °C; IR ν: 1590 (CH_{Ar}), 1360, 1155 (SO_2), 550 (CBr_3) cm^{-1}; ^1H NMR (400 MHz, $CDCl_3$) δ 8.12–7.98 (m, 2H), 7.87–7.70 (m, 2H).

4-Chlorophenylmethyl sulfide (5)
(Method B and C)

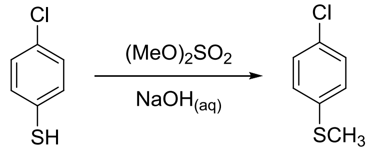

2 5

To a stirred solution of 4-chlorothiophenol (**2**) (14.2 g, 98.6 mmol) in 10% w/w aq NaOH (49.5 mL) dimethyl sulfate (25.0 g, 0.198 mol) was slowly added dropwise. When the addition was finished, 10% w/w aq NaOH was added to make the mixture alkaline (pH ~8). Next, the mixture was extracted with diethyl ether twice, and the combined organic extracts were dried over Na_2SO_4 and filtered. Ether was distilled off and the oily residue was distilled under vacuum (bp 70–72 °C / 2.66 × 10^{-3} bar). Sulfide **5** was obtained in 94% yield (14.6 g, 92.4 mmol).

4-Chlorophenyl methyl sulfone (4)
(Method B and C)

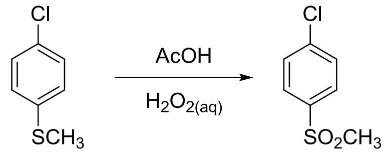

5 4

After heating the mixture of sulfide **5** (20.15 g, 0.1275 mol) and glacial acetic acid (95 mL) to its boiling point, 30% w/w aq hydrogen peroxide (40 mL) was slowly added dropwise. The mixture was refluxed for 3 hours, and afterwards the contents of the flask were poured onto ice. The precipitate was filtered off and washed with cold water until the filtrate became neutral. Recrystallization from ethanol afforded the product **4** as a white crystalline solid (23.25 g, 0.1224 mol, 96%); mp 97–98 °C.

4-Chlorophenyl tribromomethyl sulfone (1)
(Method B)

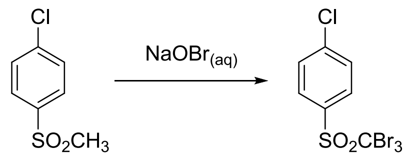

4 **1**

4-Chlorophenyl methyl sulfone **4** (9.5 g, 0.05 mol) was added to freshly prepared 14% w/w aq sodium hypobromite (150 mL), and the resulting mixture was stirred at 75 °C for 3 hours. After cooling the mixture to ambient temperature, the precipitate was filtered off, washed with water, dried and recrystallized from 2-propanol. Product **1** was obtained in 95% yield (20.3 g, 47.5 mmol); mp 165–167 °C; IR ν: 1590 (CH_{Ar}), 1335, 1125 (SO_2), 550 (CBr_3) cm^{-1}; ^1H NMR (400 MHz, $CDCl_3$) δ 8.02–7.95 (m, 2H), 7.67– 7.60 (m, 2H).

4-Halogenphenyltribromomethylsulfone 1 and 1'
(Method C)

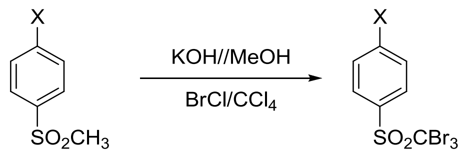

4 X = Cl
4' X = Br
1 X = Cl
1' X = Br

To a solution of KOH (2.8 g, 0.050 mol) in methanol (30 mL), 4-halogenphenyl methyl sulfone **4** (1.91 g, 0.01 mol) or **4'** (2.35 g, 0.01 mol) was added. After the stirred mixture was cooled below 20 °C, bromine chloride (3.0 g of a 50% w/w solution in carbon tetrachloride, 0.013 mol) was added dropwise. The mixture was stirred at room temperature for 2 hours. The precipitate was filtered off, washed with hexane, dried and recrystallized from 2-propanol. Sulfone **1** was obtained in 94% yield (4.02 g, 9.41 mmol); mp 165–167 °C.

Sulfone **1'** was obtained in 90% yield (4.25 g, 9.01 mmol); mp 174–175 °C; ^{1}H NMR (400 MHz, CDCl$_3$) δ 7.99–7.92 (m, 2H), 7.62–7.56 (m, 2H).

4-Halogeno-3-nitrophenyltribromomethylsulfone 6 and 6'

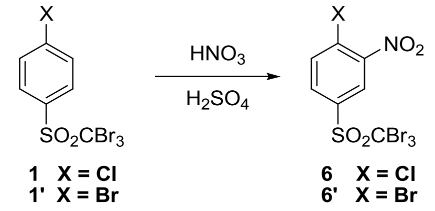

Sulfone **1** (8.55 g, 0.02 mol) or **1'** (9.44 g, 0.02 mol) was dissolved in concentrated (min. 95% w/w) sulfuric acid (25 mL). The mixture was heated to 60 °C and concentrated (65% w/w) nitric acid (1.7 mL, 0.025 mol HNO_3) was added slowly with the temperature kept below 70 °C. When the addition was finished, the mixture was heated at 80 °C for 2 hours. Then, the mixture was cooled down and poured onto crushed ice. The precipitate was filtered off, washed with water and dried. The product was purified by recrystallization from 2-propanol. Nitrophenyl sulfone **6** was obtained in 96% yield (9.08 g); mp 172–173 °C; ^{1}H NMR (400 MHz, CDCl$_3$) δ 8.42 (dd, 1H), 8.20–8.12 (m, 2H). Nitrosulfone **6'** was obtained in 94% yield (9.72 g); mp 178–180 °C; ^{1}H NMR (400 MHz, CDCl$_3$) δ 8.34 (dd, 1H), 8.16–8.10 (m, 2H).

2-Nitro-4-tribromomethylsulfonylaniline 7a

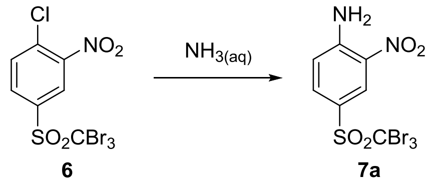

To a solution of nitrosulfone **6** (23.6 g, 0.05 mol) in dioxane (200 mL) 25% w/w aqueous ammonia (100 mL) was added. After stirring the mixture for two hours at 60 °C, another portion of 25% w/w aqueous ammonia (50 mL) was added. The reaction mixture was maintained for another two hours at 60 °C. The mixture was cooled down to room temperature and concentrated in vacuo. The precipitate was filtered off, washed with water, dried and recrystallized from ethanol. Product 7a was obtained in 95% yield (21.6 g, 47.7 mmol); mp 273–275 °C.

2-Nitro-4-tribromomethylsulfonylphenylhydrazine (7b)

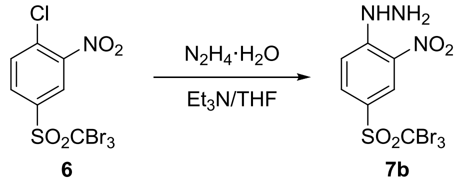

Nitrosulfone **6** (4.72 g, 0.01 mol) and triethylamine (1.4 mL, 0.01 mol) was dissolved in tetrahydrofurane (20 mL). Hydrazine hydrate (40% w/w N_2H_4, 1.5 mL, ca 0.05 mmol) was slowly added, with the temperature kept below 20 °C. The resulting mixture was stirred for 1.5 hours at room temperature. The solvent was evaporated and the precipitate dissolved in dichloromethane (50 mL). The solution was consecutively washed with 10% w/w aq HCl, water, saturated aq $NaHCO_3$ and water. The organic layer was dried over $MgSO_4$, filtered and concentrated in vacuo. Recrystallization from ethanol afforded product **7b** (4.41 g, 9.43 mmol, 94% yield); mp 218–220 °C.

2-Nitroaniline derivatives (7c–7h)
(General procedure)

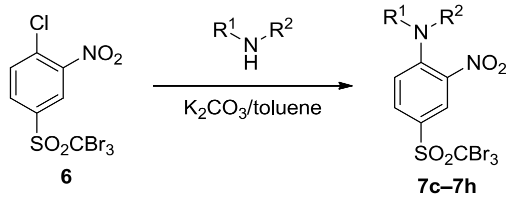

structures of compounds **7c–7h** are given in Table 1

Nitrosulfone **6** (4.72 g, 0.01 mol), the appropriate nonaromatic amine (0.01 mol) and potassium carbonate (2 g, 0.015 mmol) were mixed in toluene (30 mL). The mixture was heated under reflux for 6 hours. After cooling, diethyl ether (50 mL) was added. The mixture was consecutively washed with 10% w/w aq HCl, water, saturated aq $NaHCO_3$ and water. The organic layer was dried over $MgSO_4$, filtered and concentrated in vacuo. The product was recrystallized from 2-propanol.

2-Nitroaniline derivatives (7i–7k)

(General procedure)

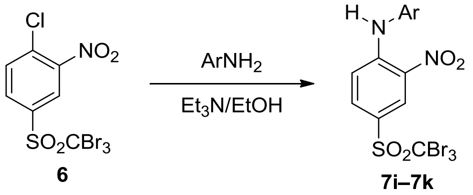

structures of compounds **7i–7k** are given in Table 1

Nitrosulfone **6** (4.72 g, 0.01 mol), the appropriate aromatic amine (0.01 mol) and triethylamine (1.4 mL, 0.01 mol) were dissolved in ethanol (35 mL). The mixture was heated under reflux for 6 hours. After cooling, ethanol was evaporated under reduced pressure, and the precipitate was dissolved in dichloromethane (40 mL). The mixture was consecutively washed with 10% w/w aq HCl, water, saturated aq $NaHCO_3$ and water. The organic layer was dried over $MgSO_4$, filtered and concentrated in vacuo. The product was recrystallized from 2-propanol.

Diphenyl ether derivatives (7l–7o)

(General procedure)

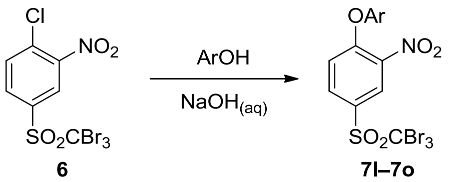

structures of compounds **7l–7o** are given in Table 1

Nitrosulfone **6** (4.72 g, 0.01 mol), appropriate phenol (0.01 mol) and 10% w/w aqueous sodium hydroxide (50 mL) was stirred and heated under reflux for 3 hours. After cooling to room temperature, the precipitate was filtered off, washed with water, dried and recrystallized from 2-propanol or ethanol.

2-Nitro-4-tribromomethylsulfonylphenylhydrazones (8a–8l)
(General procedure)

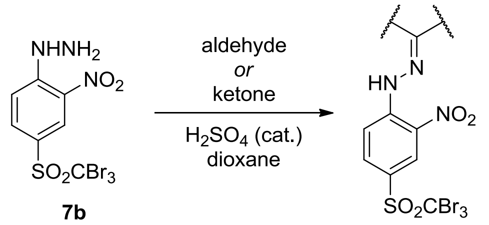

7b 8a–8l

structures of compounds **8a–8l** are given in Table 2

To the solution of 2-nitro-4-(tribromomethylsulfonyl)phenylhydrazine (**7b**) (4.68 g, 0.01 mol) in dioxane (15 mL), concentrated (min. 95% w/w) sulfuric acid (0.2 mL) was added dropwise. The mixture was stirred for a few minutes at room temperature and the appropriate ketone or aldehyde (0.01 mol) was added. After stirring for 30 minutes, the precipitate was filtered off, washed with water (until the water after filtration achieved a pH of 7), dried and recrystallized from 2-propanol.

4-Tribromomethylsulfonyl-1,2-phenylenediamine (9)

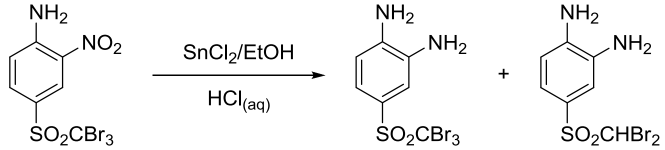

7a 9 10

The stirred mixture of 2-nitro-4-(tribromomethylsulfonyl)aniline (**7a**) (22.65 g, 0.05 mol), anhydrous stannous chloride (66.3 g, 0.35 mol) and ethanol (200 mL) was heated to 70 °C. Then 250 mL of concentrated (35–38% w/w) hydrochloric acid was slowly added dropwise, with the temperature kept within the range of 70–75 °C. After cooling to room temperature, the precipitate was filtered off and treated with 15% w/w aqueous sodium hydroxide. The resulting residue was separated, washed with water, dried and recrystallized from ethanol. Phenylenediamine **9** was isolated in 60% yield (12.7 g, 0.03 mol); mp 164–165 °C. After concentrating and cooling the filtrate, pale yellow crystals of 4-dibromomethylsulfonyl-1,2-phenylenediamine (**10**) were obtained in 30% yield (5.2 g, 0.015 mol); mp 148–150 °C.

5-Tribromomethylsulfonylbenzimidazole derivatives (11a–11e)
(General procedure)

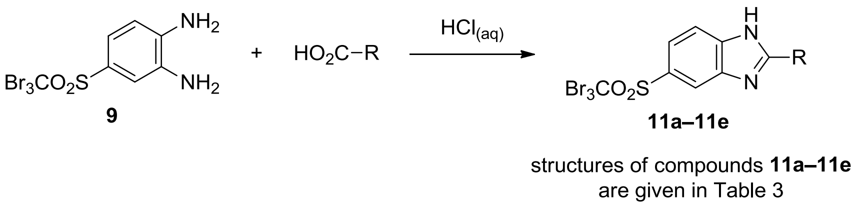

The suspension of 4-tribromomethylsulfonyl-1,2-phenylenediamine (**9**) (4.23 g, 0.01 mol) and the appropriate carboxylic acid (0.01 mol) in 4 M aqueous hydrochloric acid (30 mL) was stirred and heated under reflux for three hours (or six hours, in the case of the preparation of **11d** and **11e**). The suspension was neutralized by aqueous ammonia, filtered off, washed with water, dried and recrystallized from ethanol.

5-Tribromomethylsulfonylbenzimidazole derivatives (11f–11g)
(General procedure)

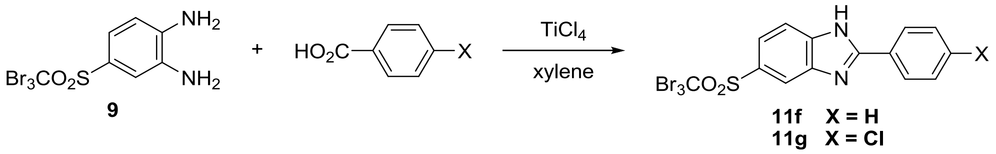

To a solution of the appropriate carboxylic acid (0.01 mol) in dry xylene (20 mL), titanium tetrachloride (1.1 mL, 0.01 mol) was added under a nitrogen atmosphere. The solution was stirred and diamine **9** (4.23 g, 0.01 mol) was added. The reaction mixture was heated at 130 °C for 2 hours. After cooling to room temperature, the reaction mixture was neutralized with a saturated aqueous solution of sodium bicarbonate. The layers were separated and the aqueous layer was extracted with ethyl acetate. The combined organic extracts were washed with water, dried over Na_2SO_4 and concentrated in vacuo. The residue was recrystallized from ethanol.

5-Tribromomethylsulfonyl-2-mercaptobenzimidazole (11h)

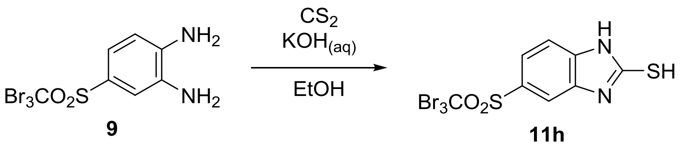

A mixture of 4-tribromomethylsulfonyl-1,2-phenylenediamine (**9**) (4.23 g, 0.01 mol), carbon disulfide (1.14 g, 0.015 mol), potassium hydroxide (0.84 g, 0.015 mol) in water (10 mL) and ethanol (20 mL) was heated under reflux for 5 hours. The reaction mixture was concentrated under reduced pressure and water (20 mL) was added to the residue. The mixture was acidified with acetic acid. The precipitate was separated, washed with water and recrystallized from ethanol. Mercaptobenzimidazole **11h** was obtained in 80% yield (3.72 g, 8 mmol); mp 276–278 °C.

Benzimidazolethiocarbamate derivatives (11i-11j)
(General procedure)

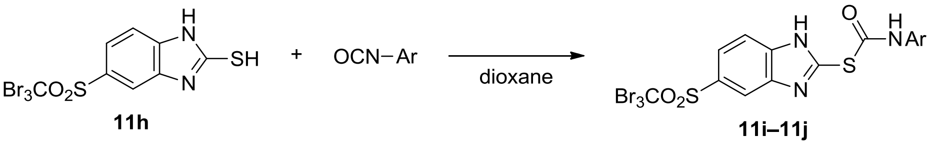

structures of compounds **11i–11j** are given in Table 3

A mixture of 5-tribromomethylsulfonyl-2-mercaptobenzimidazole (**11h**) (0.01 mol) and the appropriate isocyanate (0.01 mol) in dry dioxane (15 mL) was heated under reflux for 3 hours. After cooling the reaction mixture to ambient temperature, the precipitate was filtered off and purified by washing with hot chloroform.

Table 1. Derivatives of 2-nitroaniline, 2-nitrophenylhydrazine and diphenyl ether.

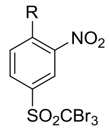

Comp. No.	R	Mol. Formula	Mol. Wt.	M.P.	Yield	Elemental analyses						IR
			[g/mol]	[°C]	[%]	Calcd.			Found			cm^{-1}
						% C	% H	%N	% C	% H	% N	
**7a**	NH_2	$C_7H_5Br_3N_2O_4S$	452.90	273-275	93	18.56	1.11	6.19	18.38	1.06	6.12	NH_2 3450, 3350,NO_2 1540, 1370, SO_2 1340, 1160, C-Br_3 540
**7b**	$NHNH_2$	$C_7H_6Br_3N_3O_4S$	468.92	218-220	94	17.93	1.50	8.96	17.81	1.38	8.78	NH 3380, 3325, NO_2 1555, 1365, SO_2 1345,1160, C-Br_3 540
**7c**	$NHCH_3$	$C_8H_7Br_3N_2O_4S$	466.93	228-230	95	20.58	1.51	6.00	20.68	1.61	5.94	NH 3360, NO_2 1565, 1370, SO_2 1355,1150, C-Br_3 550
**7d**	NHC_6H_{11}	$C_{13}H_{15}Br_3N_2O_4S$	535.05	194-195	87	29.18	2.83	5.24	29.26	2.91	5.15	NH 3360, NO_2 1530, 1370, SO_2 1355,1160, C-Br_3 550
**7e**	$NHCH_2CH_2CH_3$	$C_{10}H_{11}Br_3N_2O_4S$	494.98	185-186	92	24.27	2.24	5.66	33.34	3.12	7.65	NH 3370, NO_2 1555, 1365, SO_2 1345,1160, C-Br_3 550
**7f**	$NHCH_2CH(CH_3)_2$	$C_{11}H_{13}Br_3N_2O_4S$	509.01	154-135	94	25.96	2.57	5.50	25.75	2.64	5.40	NH 3350, NO_2 1555, 1365, SO_2 1355,1150, C-Br_3 550
**7g**	$N(C_2H_5)_2$	$C_{11}H_{13}Br_3N_2O_4S$	510.02	175-176	94	25.91	2.77	5.49	25.76	3.02	5.28	NO_2 1560, 1360, SO_2 1345,1150, C-Br_3 545
**7h**	$N(CH_2CH(CH_3)_2)_2$	$C_{15}H_{21}Br_3N_2O_4S$	565.11	147-149	92	31.88	3.75	4.96	31.94	3.58	4.87	NO_2 1555, 1365, SO_2 1350,1160, C-Br_3 550
**7i**	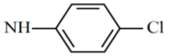	$C_{13}H_8ClBr_3N_2O_4S$	563.44	196-197	96	27.71	1.43	4.97	27.62	1.51	4.78	NH 3345, NO_2 1560, 1360, SO_2 1345,1155, C-Br_3 540
**7j**	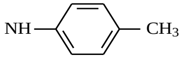	$C_{14}H_{11}Br_3N_2O_4S$	543.02	184-186	88	30.97	2.04	5.16	31.07	1.97	5.06	NH 3330, NO_2 1520, 1380, SO_2 1350,1175, C-Br_3 550
**7k**	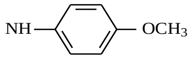	$C_{14}H_{11}Br_3N_2O_5S$	559.02	173-174	86	30.08	1.98	5.01	30.01	1.84	4.95	NH 3330, NO_2 1540, 1360, SO_2 1350,1160, C-Br_3 555
**7l**	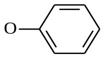	$C_{13}H_8Br_3NO_5S$	529.98	144-145	91	29.46	1.52	2.64	29.64	1.73	2.48	NO_2 1555, 1365, SO_2 1355,1150, C-O-C 1250, C-Br_3 550
**7m**	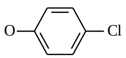	$C_{13}H_7ClBr_3NO_5S$	564.43	148-149	92	27.66	1.25	2.48	27.85	1.37	2.39	NO_2 1550, 1365, SO_2 1355,1155, C-O-C 1250, C-Br_3 550
**7n**	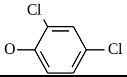	$C_{13}H_6Cl_2Br_3NO_5S$	598.87	154-156	92	26.07	1.01	2.34	26.32	1.12	2.26	NO_2 1550, 1365, SO_2 1360,1160, C-O-C 1250, C-Br_3 550
**7o**	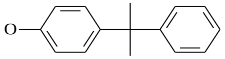	$C_{22}H_{18}Br_3NO_5S$	648.16	167-168	90	40.77	2.80	2.16	40.65	2.55	2.08	NO_2 1550, 1365, SO_2 1355,1155, C-O-C 1260, C-Br_3 550

Table 2. 2-Nitro-4-tribromomethylsulfonylphenylhydrazones.

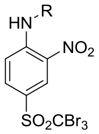

Comp. No	R	Mol. Formula	Mol. Wt. [g/mol]	M.P. [°C]	Yield [%]	Elemental analyses: Calcd. % C	Calcd. % H	Calcd. %N	Found % C	Found % H	Found % N	IR cm^{-1}
**8a**	$N=CCH_3C_3H_7$	$C_{12}H_{14}Br_3N_3O_4S$	536.03	168-170	96	26.89	2.63	7.84	26.94	2.55	7.75	NH 3330, NO_2 1555, 1350, SO_2 1355,1165, C-Br_3 550
**8b**	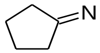	$C_{12}H_{12}Br_3N_3O_4S$	534.02	197-199	87	26.99	2.26	7.89	26.56	2.08	7.75	NH 3320, NO_2 1550, 1350, SO_2 1355,1160, C-Br_3 555
**8c**	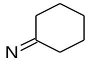	$C_{13}H_{14}Br_3N_3O_4S$	548.04	212-214	89	28.49	2.57	7.67	28.38	2.38	7.56	NH 3330, NO_2 1550, 1350, SO_2 1355,1160, C-Br_3 555
**8d**	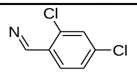	$C_{14}H_8Br_3Cl_2N_3O_4S$	624.91	225-227	93	26.91	1.29	6.72	26.76	1.32	6.64	NH 3335, NO_2 1550, 1350, SO_2 1355,1160, C-Br_3 550
**8e**	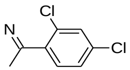	$C_{15}H_{10}Br_3Cl_2N_3O_4S$	638.94	174-175	90	28.20	1.58	6.58	28.31	1.46	6.44	NH 3335, NO_2 1550, 1350, SO_2 1355,1155, C-Br_3 550
**8f**	$N=C(C_6H_5)_2$	$C_{20}H_{14}Br_3N_3O_4S$	632.12	243-244	92	38.00	2.23	6.65	37.87	2.18	6.56	NH 3335, NO_2 1550, 1350, SO_2 1350,1155, C-Br_3 550
**8g**	$N=CHCH_3$	$C_9H_8Br_3N_3O_4S$	493.95	195-197	85	21.88	1.63	8.51	21.76	1.45	8.38	NH 3325, NO_2 1555, 1350, SO_2 1350,1155, C-Br_3 555
**8h**	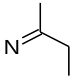	$C_{11}H_{12}Br_3N_3O_4S$	522.01	181-182	89	25.31	2.32	8.05	25.12	2.24	7.98	NH 3325, NO_2 1555, 1350, SO_2 1350,1155, C-Br_3 550
**8i**	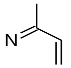	$C_{11}H_{10}Cl_3N_3O_4S$	519.99	202-204	91	25.41	1.94	8.08	25.33	1.76	7.97	NH 3325, NO_2 1550, 1350, SO_2 1350,1165, C-Br_3 555
**8j**	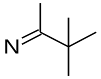	$C_{13}H_{16}Br_3N_3O_4S$	550.06	144-145	88	28.39	2.93	7.64	28.43	2.78	7.57	NH 3325, NO_2 1555, 1350, SO_2 1350,1155, C-Br_3 550
**8k**	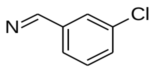	$C_{14}H_9Br_3ClN_3O_4S$	590.47	284-286	92	28.48	1.54	7.12	28.37	1.44	7.04	NH 3325, NO_2 1550, 1350, SO_2 1350,1165, C-Br_3 550
**8l**	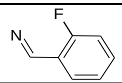	$C_{14}H_9Br_3FN_3O_4S$	574.01	246-247	94	29.29	1.58	7.32	29.08	1.48	7.24	NH 3325, NO_2 1565, 1350, SO_2 1350,1155, C-Br_3 550

Table 3. 5-Tribromomethylsulfonylbenzimidazole derivatives.

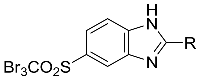

Comp. No.	R	Mol. Formula	Mol. Wt.	M.P.	Yield	Elemental analyses						IR
			[g/mol]	[°C]	[%]	Calcd.			Found			cm^{-1}
						% C	% H	%N	% C	% H	% N	
**11a**	CH_3	$C_9H_7Br_3N_2O_2S$	446.94	187-189	86	24.19	1.58	6.27	24.34	1.72	6.18	NH 3330, SO_2 1335,1130, CBr_3 550
**11b**	CF_3	$C_9H_4Br_3F_3N_2O_2S$	500.91	241-243	93	21.58	0.80	5.59	22.12	0.88	5.46	NH 3300, SO_2 1330,1120, CBr_3 555
**11c**	$CH_2C_6H_5$	$C_{15}H_{11}Br_3N_2O_2S$	523.04	234-235	57	34.45	2.12	5.36	34.25	1.97	5.27	NH 3360, SO_2 1335,1130, CBr_3 550
**11d**	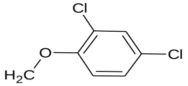	$C_{15}H_9Br_2Cl_2N_2O_3S$	607.93	238-239	81	29.64	1.49	4.61	29.45	1.37	4.43	NH 3350, SO_2 1325,1145, CBr_3 550
**11e**	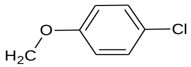	$C_{15}H_{10}Br_3ClN_2O_3S$	573.48	227-229	77	31.42	1.76	4.88	31.30	1.55	4.76	NH 3330, SO_2 1330, 1140, CBr_3 555
**11f**	C_6H_5	$C_{14}H_9Br_3N_2O_2S$	509.01	267-269	79	33.04	1.78	5.50	32.78	1.57	5.38	NH 3300, SO_2 1330,1125, CBr_3 550
**11g**	C_6H_4-4-Cl	$C_{11}H_{13}Cl_3N_4O_3S$	385.65	270-272	65	34.26	2.87	14.53	34.40	2.92	14.46	NH 3360, SO_2 1335,1155, CBr_3 550
**11h**	SH	$C_8H_5Br_3N_2O_2S_2$	464.97	276-278	80	20.67	1.08	6.02	20.46	1.15	5.94	NH 3400, SO_2 1335, 1150, CBr_3 555
**11i**	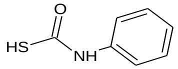	$C_{15}H_{10}Br_3N_3O_3S_2$	584.09	340 (d)	92	30.85	1.73	7.19	30.62	1.56	7.12	NH 3350, C=O 1715, SO_2 1340, 1150, CBr_3 555
**11j**	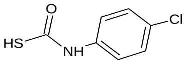	$C_{15}H_9Br_3N_3O_3S_2$	618.54	325-326	76	29.13	1.47	6.79	29.02	1.26	6.74	NH 3360, C=O 1710, SO_2 1340, 1150, CBr_3 550

d - decomposition

BrB7f

Pulse Sequence: s2pul
Solvent: CDCl3
Ambient temperature
Mercury-400BB "mercury"

PULSE SEQUENCE
Pulse 37.5 degrees
Acq. time 4.000 sec
Width 7400.0 Hz
44 repetitions
OBSERVE H1, 400.1050225 MHz
DATA PROCESSING
FT size 65536
Total time 36 min, 42 sec

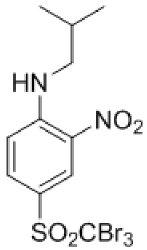

7f

^1H NMR

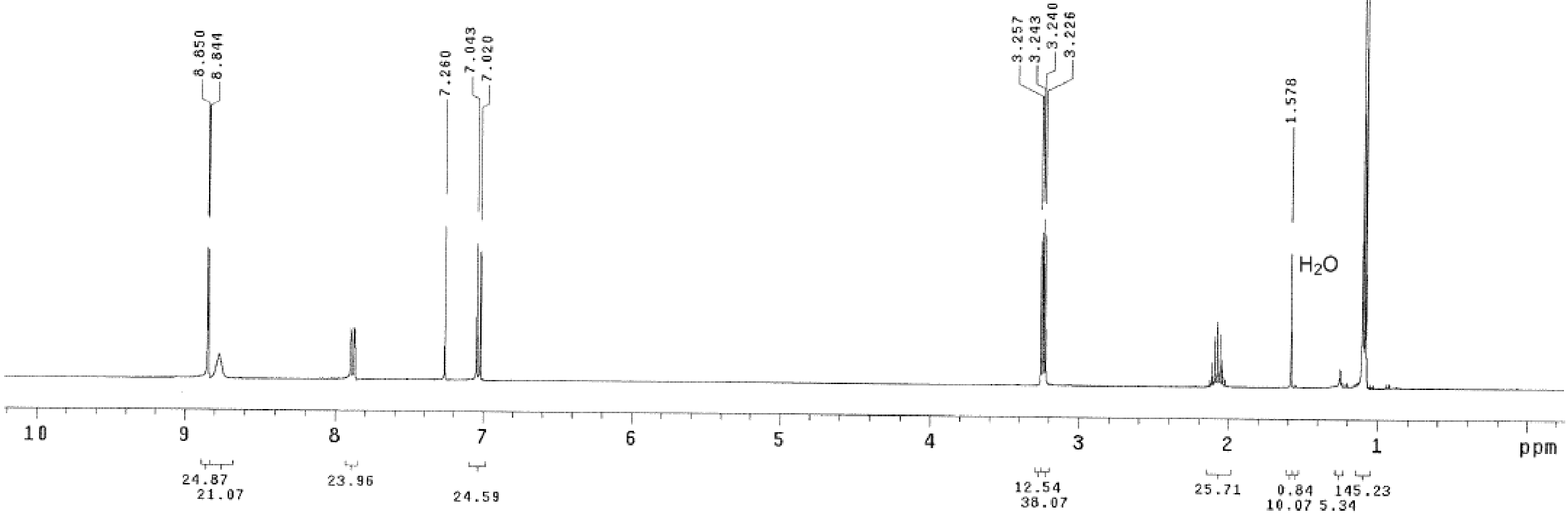

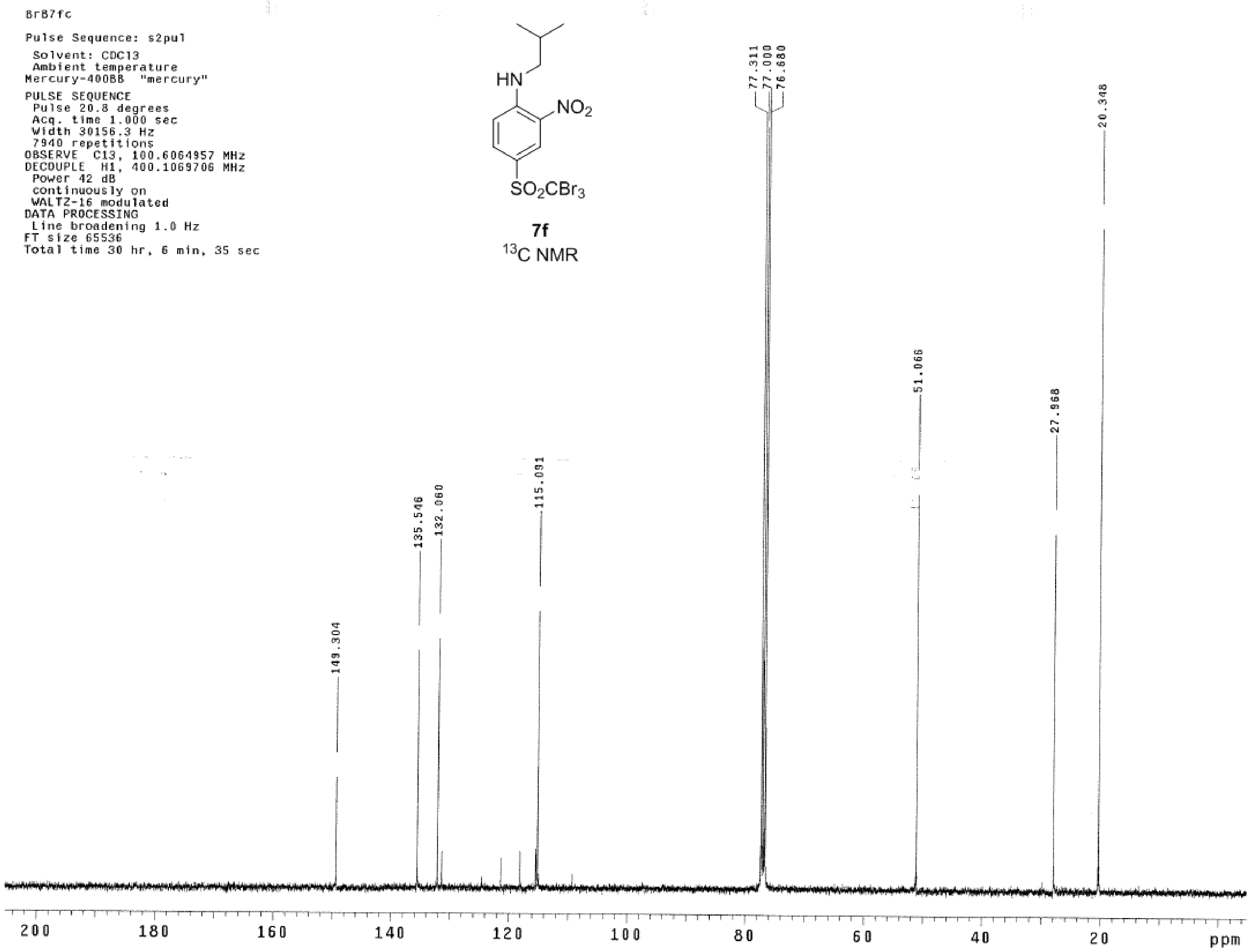
BrB7fc
Pulse Sequence: s2pul
Solvent: CDCl3
Ambient temperature
Mercury-400BB "mercury"
PULSE SEQUENCE
Pulse 20.8 degrees
Acq. time 1.000 sec
Width 30156.3 Hz
7940 repetitions
OBSERVE C13, 100.6064957 MHz
DECOUPLE H1, 400.1069706 MHz
Power 42 dB
continuously on
WALTZ-16 modulated
DATA PROCESSING
Line broadening 1.0 Hz
FT size 65536
Total time 30 hr, 6 min, 35 sec
HN
NO2
SO2CBr3
7f
13C NMR
149.304
135.546
132.060
115.091
77.311
77.000
76.680
51.066
27.968
20.348
200
180
160
140
120
100
80
60
40
20
ppm

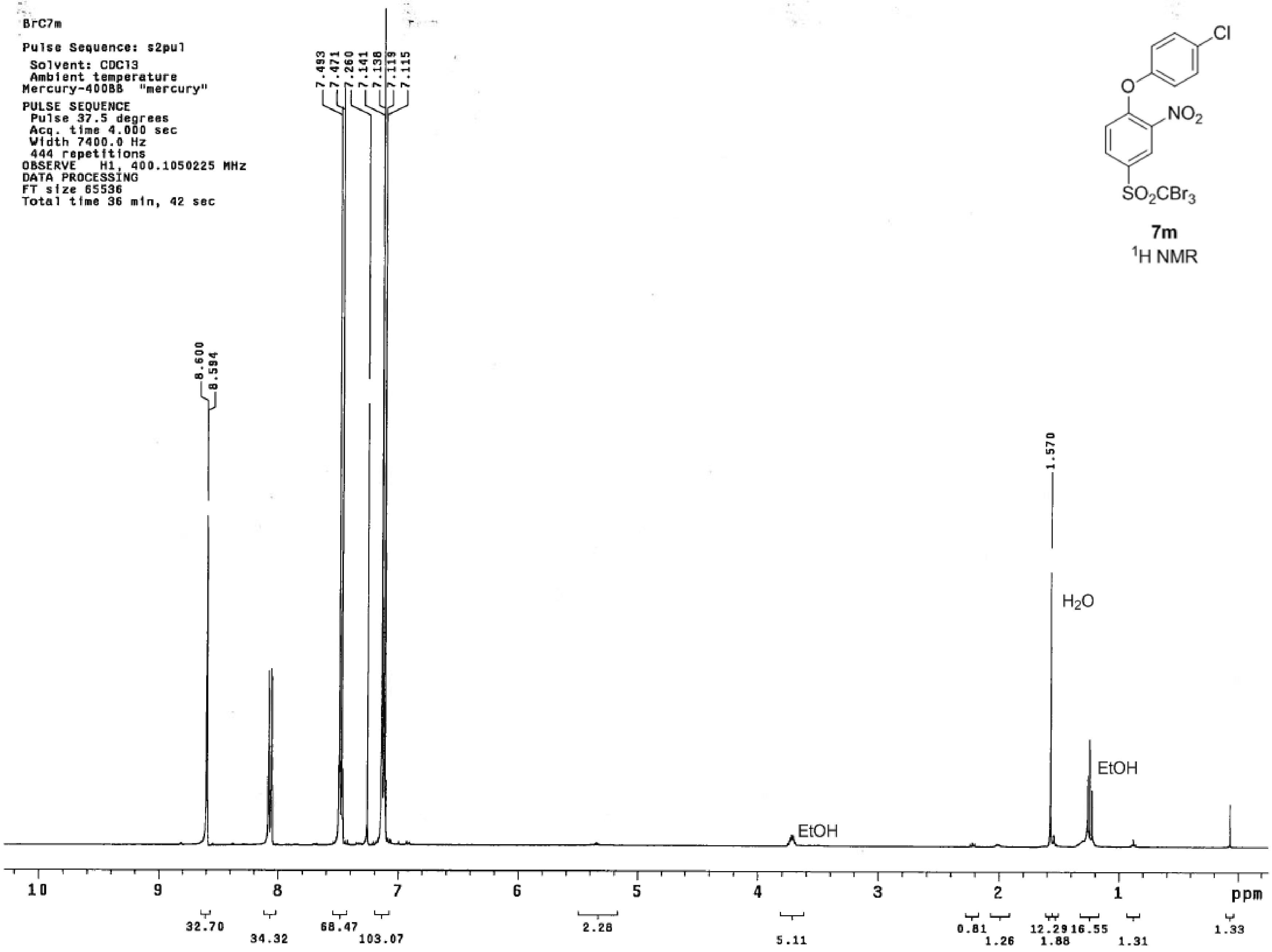
BrC7m
Pulse Sequence: s2pul
Solvent: CDCl3
Ambient temperature
Mercury-400BB "mercury"
PULSE SEQUENCE
Pulse 37.5 degrees
Acq. time 4.000 sec
Width 7400.0 Hz
444 repetitions
OBSERVE H1, 400.1050225 MHz
DATA PROCESSING
FT size 65536
Total time 36 min, 42 sec
7.493
7.471
7.260
7.141
7.138
7.119
7.115
8.600
8.594
1.570
H2O
EtOH
EtOH
10
9
8
7
6
5
4
3
2
1
ppm
32.70
34.32
68.47
103.07
2.28
5.11
0.81
1.26
12.29
1.88
16.55
1.31
1.33
Cl
O
NO2
SO2CBr3

7m
^1H NMR

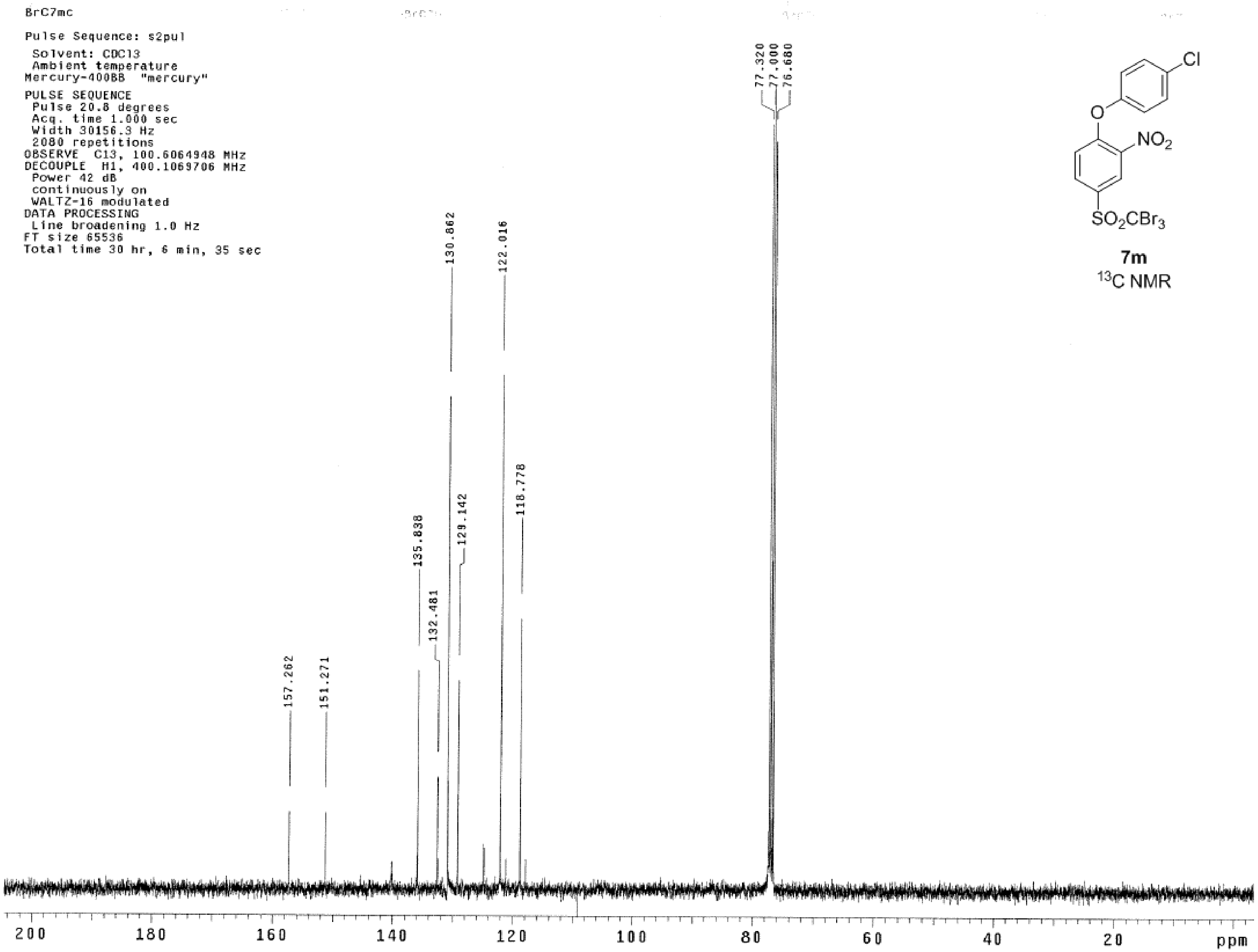
BrC7mc
Pulse Sequence: s2pul
Solvent: CDCl3
Ambient temperature
Mercury-400BB "mercury"
PULSE SEQUENCE
Pulse 20.8 degrees
Acq. time 1.000 sec
Width 30156.3 Hz
2080 repetitions
OBSERVE C13, 100.6064948 MHz
DECOUPLE H1, 400.1069706 MHz
Power 42 dB
continuously on
WALTZ-16 modulated
DATA PROCESSING
Line broadening 1.0 Hz
FT size 65536
Total time 30 hr, 6 min, 35 sec
77.320
77.000
76.680
130.862
122.016
118.778
129.142
135.838
132.481
157.262
151.271
200
180
160
140
120
100
80
60
40
20
ppm
Cl
O
NO2
SO2CBr3

7m

^{13}C NMR
